# Efficacy and safety of acupuncture in the treatment of postherpetic neuralgia: A protocol for systematic review and network meta-analysis

**DOI:** 10.1097/MD.0000000000027088

**Published:** 2021-09-10

**Authors:** Jian Zhao, Zhongguang Zhou, Xin He, Yin Yuan, Di Wang

**Affiliations:** a Basic Medical School, Heilongjiang University of Chinese Medicine, Harbin, Heilongjiang Province, China; b Second Affiliated Hospital of Heilongjiang University of Chinese Medicine, Harbin, Heilongjiang Province, China.

**Keywords:** acupuncture, network meta-analysis, postherpetic neuralgia, protocol

## Abstract

**Background:**

It is simple, convenient, inexpensive, proven, extensive, and safe for acupuncture in the treatment of postherpetic neuralgia (PHN). However, there are no comparisons between various acupuncture therapies that can directly and effectively provide specific guidance to clinicians. The development of a guideline for the optimization of acupuncture for PHN is of great importance for the development of clinical acupuncture. Therefore, we attempted to design a study protocol for a network meta-analysis of randomized controlled trials of acupuncture for PHN to provide evidence to support the treatment of acupuncture for PHN.

**Methods:**

A search of 8 databases, Chinese Scientific Journal Database, China National Knowledge Infrastructure Database, Wanfang, China Biomedical Literature Database, PubMed, Cochrane Library, Embase, and Web of Science, was conducted to collect randomized controlled trials about acupuncture for PHN. RevMan 5.3 and Stata 14.0 software were used for data analysis.

**Results:**

This meta-analysis will provide additional and more robust evidence for acupuncture treatment of PHN. Our findings will assist clinicians in making treatment decisions.

**Conclusion:**

This study will provide comprehensive and reliable evidence-based evidence for the treatment of PHN with acupuncture.

## 1 Introduction

Herpes zoster is a disease in which the varicella-zoster virus latent in sensory ganglia is reactivated and spreads from a single dorsal root ganglion to the corresponding skin and nerve tissue of the same segment, causing a severe painful rash in the corresponding skin.^[1,2]^ The main complication of herpes zoster is a severe chronic neuropathic pain called postherpetic neuralgia (PHN).^[3]^ The disease is most prevalent in middle-aged and elderly people over 50 years of age, with a prevalence of 20%.^[4]^ PHN is not life-threatening, but it has a huge impact on quality of life and is prone to recurrence, which may cause serious physical and psychological damage to the patient.

Current research suggests that the pathogenesis of PHN is mainly due to the sequelae of the varicella-zoster virus invading the body and the virus in the patient's body and the virus transmitted to the peripheral nerves not being cleared out of the body, causing an inflammatory response by damaging the peripheral nerves, leading to nerve ischemia and hypoxia, which in turn leads to demyelinating changes in the nerves and loss of myelin protection, resulting in pain, lightning-like episodes of pain, and other abnormal sensations.^[5,6]^

Western medicine treats the disease mostly with antiviral, analgesic, nerve-nourishing, and hormonal drugs.^[7,8]^ Short-term symptom control and pain relief are good, but the overall efficacy is poor.^[9]^ Long-term use of such drugs is likely to cause vertigo, peripheral edema, and gastrointestinal reactions. There is no cure for the disease.

Traditional Chinese medicine refers to taking PHN as Shedan Healing Pain, and it is believed that the occurrence of the patient's disease is related to the imbalance of Ying and Wei, lack of righteousness, and obstruction of the meridian.^[10–12]^ Therefore, the treatment for patients is mainly to clear the meridian and activate the collaterals, remove blood stasis and relieve pain, and drive away evil and strengthen the body. The treatment methods of Chinese medicine for this disease mainly include acupuncture, moxibustion, acupuncture cupping, medicinal thread, Chinese herbal medicine, external application, and scrubbing.^[13,14]^ Among them, acupuncture has the advantages of fast efficacy, high safety, and small adverse reactions, and is the preferred treatment option for most patients in clinical practice.^[15,16]^

Clinical studies^[17–22]^ have reported good efficacy of various acupuncture protocols for PHN, but there is no comparison between various acupuncture therapies that can directly and effectively provide specific guidance for clinicians. The development of a guideline to optimize acupuncture for the treatment of PHD is of great importance for the development of clinical acupuncture. Therefore, we attempted to design a study protocol to conduct a network meta-analysis of randomized controlled trials (RCTs) of acupuncture for PHN to provide evidence to support non-pharmacological interventions for PHN.

## 2 Methods

### 2.1 Study registration

The protocol of this review will be registered in OSF Registries (OSF registration number: DOI 10.17605/OSF.IO/R4P9Z), which follows the statement guidelines of preferred reporting items for systematic reviews and meta-analyses protocol.^[23]^

### 2.2 Inclusion criteria for study selection

1. Study type: RCTs;
2. Participants: Patients definitively diagnosed with PHN, regardless of gender, age, race, or geographic location;
3. Interventions: Treatment group: acupuncture therapies, including acupuncture, warm acupuncture, head acupuncture, auricular acupuncture, electroacupuncture, acupuncture point embedding, acupuncture point dressing, acupuncture point injection, etc, may be supplemented with other therapies; Control group: drugs, placebo, sham acupuncture, waiting for treatment, etc;
4. Outcome indicators: total effective rate, pain score, cure rate, clinical symptom score, time to crusting, time to pain disappearance, morbidity, time to pain relief, time to debridement, sleep quality, quality of life, anxiety and depression score, and adverse effects.

### 2.3 Exclusion criteria

1. Duplicate publications;
2. Incomplete data;
3. Studies with inconsistent outcomes;
4. Animal experiment.

### 2.4 Data sources

A search of 8 databases, Chinese Scientific Journal Database, China National Knowledge Infrastructure Database, Wanfang, China Biomedical Literature Database, PubMed, Cochrane Library, EMBASE, and Web of Science, was conducted to collect RCTs on acupuncture for PHN. MeSH terms and keywords were used. A combined search was conducted to collect as much relevant literature as possible, and references were tracked to add any literature that might have been missed. The search ran from the date of database creation until July 2021.

### 2.5 Searching strategy

A combination of MeSH terms and free words were adopted in the searching strategy. Searching strategies using the PubMed were illustrated in Table 1, and literature search in other online databases were similarly conducted.

**Table 1:** Search strategy in PubMed database.

Number	Search terms
#1	Neuralgia, Postherpetic[MeSH]
#2	Postherpetic Neuralgia[Title/Abstract]
#3	OR/1-2
#4	Acupuncture[MeSH]
#5	Acupuncture Therapy[MeSH]
#6	Therapy, Acupuncture[Title/Abstract]
#7	Acupuncture;Medicine, Chinese Traditional[MeSH]
#8	Electroacupuncture[MeSH]
#9	Acupuncture, Ear[MeSH]
#10	fire needle[Title/Abstract]
#11	warm acupuncture[Title/Abstract]
#12	blood-pricking[Title/Abstract]
#13	acupuncture-moxibustion[Title/Abstract]
#14	acupoint[Title/Abstract]
#15	moxa needle[Title/Abstract]
#16	auricular needle[Title/Abstract]
#17	ear acupuncture[Title/Abstract]
#18	moxibustion[Title/Abstract]
#19	abdom∗ acupuncture[Title/Abstract]
#20	embedded thread therapy[Title/Abstract]
#21	embedding thread[Title/Abstract]
#22	catgut implantation at acupoint[Title/Abstract]
#23	Acupuncture Points[MeSH]
#24	Point[Title/Abstract]
#25	OR/4-24
#26	Randomized Controlled Trials as Topic[MeSH]
#27	Clinical Trials, Randomized[Title/Abstract]
#28	Controlled Clinical Trials, Randomized[Title/Abstract]
#29	Trials, Randomized Clinical[Title/Abstract]
#30	Random∗[Title/Abstract]
#31	OR/26-30
#39	#3 AND #25 AND #31

### 2.6 Data collection and analysis

#### 2.6.1 Literature screening and data extraction

**Figure 1:**
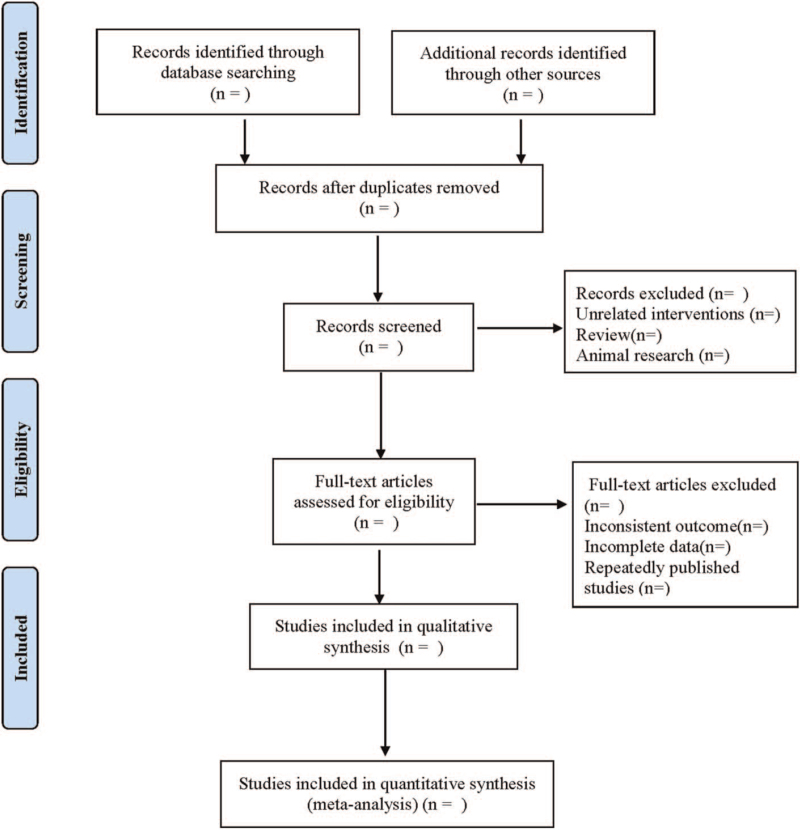
Flow diagram of study selection process.

The literature search, screening and data extraction were carried out independently by 2 researchers. In case of disagreement, the arbiter was consulted to assist in the judgment. In case of lack of information, every effort was made to contact the authors for additional information. Literature screening was performed by de-duplication through NoteExpress and then by reading the titles and abstracts of the literature to exclude those that clearly did not meet the inclusion criteria. Articles that might meet the inclusion criteria were downloaded and read in full to further clarify whether they met the inclusion criteria. Excel 2016 was used to create a table, and the extraction included information according to title, author, year of publication, number of literature, number of cases, interventions, and outcome indicators. The screening flow chart of this study was demonstrated in Figure 1.

#### 2.6.2 Assessment of evidence quality

The methodologies included in the RCTs were evaluated for quality and risk assessment using the criteria in the Cochrane Handbook 5.1.0, and risk of bias scaling maps were generated using RevMan5.3 statistical software.^[24]^ The Cochrane risk of bias assessment included 7 aspects, namely: randomization methods, blinding of participants and investigators, blinding of evaluators, allocation concealment, outcome completeness, selective reporting of results, and other sources of bias. Bias was assessed for each of the included RCTs as low risk of bias (low risk), high risk of bias (high risk), and uncertain (unclear).

#### 2.6.3 Measures of therapeutic efficacy

The dichotomous variable were estimated by relative risk, with 95% confidence intervals. Continuous variables were combined using standardized mean differences and 95% confidence intervals.

#### 2.6.4 Management of missing data

Missing data would be requested by Email; Otherwise, the data would be excluded from the study.

### 2.7 Data synthesis

#### 2.7.1 Direct comparison meta-analysis

Data were statistically analyzed using RevMan 5.3 software. Q-test with *I*^2^ test for heterogeneity of included studies, when *I*^2^ < 50% and *P* > .10, the studies were considered to have no statistical heterogeneity or less heterogeneity and a fixed-effects model could be used, and vice versa, a random-effects model was used.

#### 2.7.2 Consistency check

The Wald test was used to assess whether there was a difference between the direct and indirect evidence in the closed loop. If *P* > .05, it suggested consistency between direct and indirect evidence within the closed loop, a fitted consistency model may be used, and if not, an inconsistency model may be used and possible reasons for inconsistency explored further.

#### 2.7.3 Network meta-analysis

Network evidence plots were created using the Stata 14.0 software “networkplot” command. Network meta-analysis was performed using the “mvmeta” command in Stata 14.0 software to combine direct and indirect comparisons to produce a ranking between interventions. The surface under the cumulative ranking curves was used to rank the outcomes of the interventions, where surface under the cumulative ranking curves is an indicator of the likelihood of an intervention being better or worse, and the closer it is to 100%, the better the efficacy of the intervention.

#### 2.7.4 Assessment of reporting biases

“Comparison-adjusted” funnel plots would be depicted to evaluate publication bias.

#### 2.7.5 Subgroup analysis

Subgroup analysis would be applied based on the course of treatment.

#### 2.7.6 Sensitivity analysis

The sensitivity analysis would be performed to test the stability of the results of meta-analysis.

#### 2.7.7 Ethics and dissemination

The content of this article did not involve moral approval or ethical review and would be presented in print or at relevant conferences.

## 3 Discussion

The incidence of PHN is high and the pathogenesis is not fully understood.^[25,26]^ Current research suggests that its pathogenesis is mainly due to the sequelae of the varicella-zoster virus that is not cleared after the virus has invaded the body.^[27,28]^ At this stage, there are also relatively many studies on the mechanism of acupuncture for the treatment of PHN, but none of them have been able to fully elucidate the efficacy mechanism of acupuncture for the treatment of PHN.

High-quality meta-analyses/systematic evaluations are one of the important sources of evidence-based medicine for obtaining the best evidence and one of the best bases for clinical decision making in acupuncture. Although a large number of meta-analyses of acupuncture for PHN have been published in peer-reviewed journals, no comparisons of efficacy between various acupuncture protocols are available. In this study, we collected domestic and international RCTs of acupuncture for PHN for rigorous screening, and explored the optimal protocols of acupuncture for PHN based on network meta-analysis, with the aim of providing an evidence-based basis for the treatment of PHN. This study overcomes the shortcomings of traditional meta-analysis that cannot compare multiple interventions with each other and ranks the effects of different acupuncture methods on outcome indicators, and the results of the ranking of these interventions will help establish clinical practice guidelines for clinical decision making.

## Author contributions

**Conceptualization:** Jian Zhao, Xin He

**Data curation:** Jian Zhao, Zhongguang Zhou

**Funding acquisition:** Di Wang

**Methodology:** Jian Zhao, Xin He

**Software:** Zhongguang Zhou, Xin He

**Supervision:** Yin Yuan, Di Wang

**Writing – original draft:** Jian Zhao, Zhongguang Zhou

**Writing – review & editing:** Jian Zhao, Di Wang
